# Co-occurrence of hereditary Tyrosinemia type 1 and refractory immune thrombocytopenia: a case report

**DOI:** 10.1093/omcr/omag205

**Published:** 2026-11-02

**Authors:** Mohammad Fares Zhour, Lamia F Tayem, Saba R Zayed, Hamza S Zeedat, Mohammad Najajreh

**Affiliations:** Faculty of Medicine, Palestine Polytechnic University, Hebron 9020000, Palestine; Faculty of Medicine, Palestine Polytechnic University, Hebron 9020000, Palestine; Faculty of Medicine, Palestine Polytechnic University, Hebron 9020000, Palestine; Faculty of Medicine, Palestine Polytechnic University, Hebron 9020000, Palestine; Faculty of Medicine, Palestine Polytechnic University, Hebron 9020000, Palestine; Huda al Masri Pediatric Cancer Department, Beit Jala Hospital, Palestinian Ministry of Health, Bayt Jala, Palestine

**Keywords:** endocrinology and metabolism, haematology, Hepatology, paediatrics, gastroenterology

## Abstract

Hereditary tyrosinemia type 1 (HT-1) is a rare metabolic disorder caused by fumarylacetoacetate hydrolase deficiency. We report a unique case of HT-1 co-occurring with refractory immune thrombocytopenia (ITP) in a 3.5-year-old female. The patient initially presented at five months with severe thrombocytopenia and coagulopathy. While nitisinone therapy successfully normalized liver function and coagulation parameters, severe thrombocytopenia persisted (<10 × 109/L) despite corticosteroids, IVIG, rituximab, and thrombopoietin receptor agonists. Bone marrow examination showed normal megakaryocytes, and secondary causes were excluded. This distinct dissociation between metabolic control and hematologic refractoriness suggests a complex interplay between metabolic dysregulation and autoimmunity. This is the first reported case of such co-occurrence, highlighting the importance of considering autoimmune complications in metabolic disorders.

## Introduction

Hereditary tyrosinemia type 1 (HT-1) is a rare autosomal recessive disorder caused by fumarylacetoacetate hydrolase (FAH) deficiency, leading to accumulation of toxic metabolites like succinylacetone [1]. Clinical presentation varies from acute liver failure to renal dysfunction and neurologic crises. While nitisinone (NTBC) has revolutionized treatment [2], long-term complications remain a concern. Immune thrombocytopenia (ITP) is an autoimmune disorder characterized by platelet destruction. Refractory ITP, strictly defined according to international consensus as the failure to respond to standard first- and second-line therapies (or when splenectomy is contraindicated) with a persistent risk of clinically significant bleeding, poses profound management challenges [3]. We present a unique case of HT-1 coexisting with refractory ITP. To our knowledge, this specific co-occurrence has not been previously described, offering new insights into potential intersections between metabolic and autoimmune pathways.

## Case presentation

A 3.5-year-old female born to consanguineous parents presented at five months of age with recurrent petechiae, ecchymoses, and a 2 × 2 cm scalp hemangioma. Family history was notable for early-onset liver disease leading to death in two maternal uncles, raising suspicion of an inherited metabolic disorder. Apart from mucocutaneous bleeding, physical examination revealed hepatomegaly (liver span 8.5 cm) without splenomegaly or lymphadenopathy. Growth and developmental milestones were age-appropriate.

Initial laboratory evaluation demonstrated severe thrombocytopenia (platelet count 10–11 × 10^9^/L)) reference: 150–450 × 10^9^/L), anemia (hemoglobin 8.1 g/dL) (reference: 11.5–13.5 g/dL), and significant coagulopathy (INR 2.06; aPTT 45.5 s) (reference of INR: 0.8–1.2, aPTT: 25–35 s). Liver biochemistry showed cholestasis with alkaline phosphatase of 1749 U/L, direct hyperbilirubinemia, and markedly elevated alpha-fetoprotein (AFP) (115 786 ng/mL). Although CMV PCR testing was positive in blood and urine, the chronic nature of the presentation, absence of active end-organ viral disease (e.g. retinitis, pneumonitis), and lack of systemic inflammatory signs strongly suggested incidental latent shedding rather than an active CMV-induced myelosuppression. Abdominal CT confirmed a heterogeneous liver with innumerable hypodense lesions (Fig. 1) and a horseshoe kidney (an incidental congenital anomaly unrelated to the primary pathophysiology, yet highlighting the complexity of the patient’s presentation) (Fig. 2).

**Figure 1:**
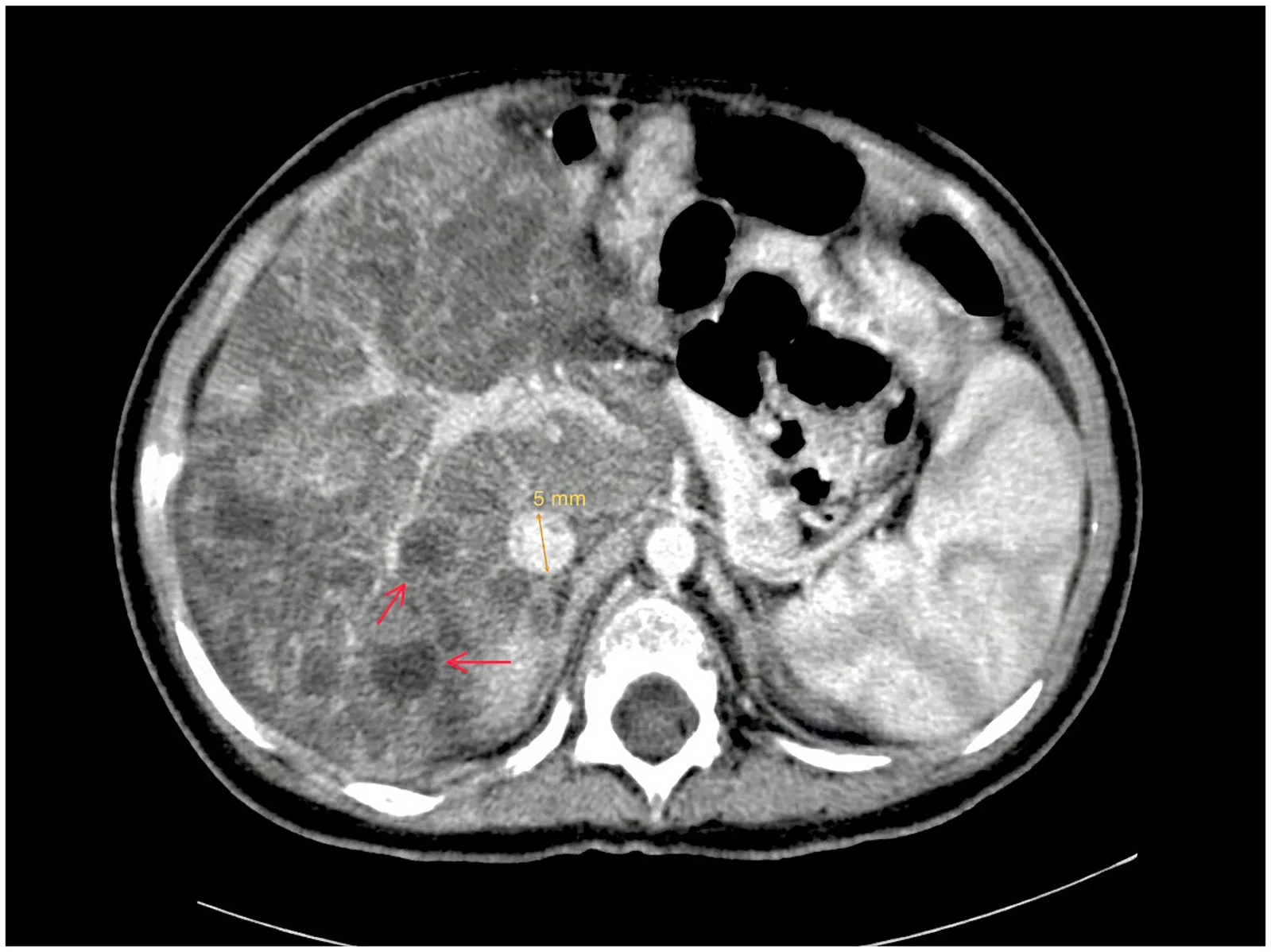
Axial CT scan of the liver showing a coarse, irregular hepatic outline with multiple hypodense lesions (indicated by arrows). The marker identifies a suspected 5 mm hepatic artery aneurysm.

**Figure 2:**
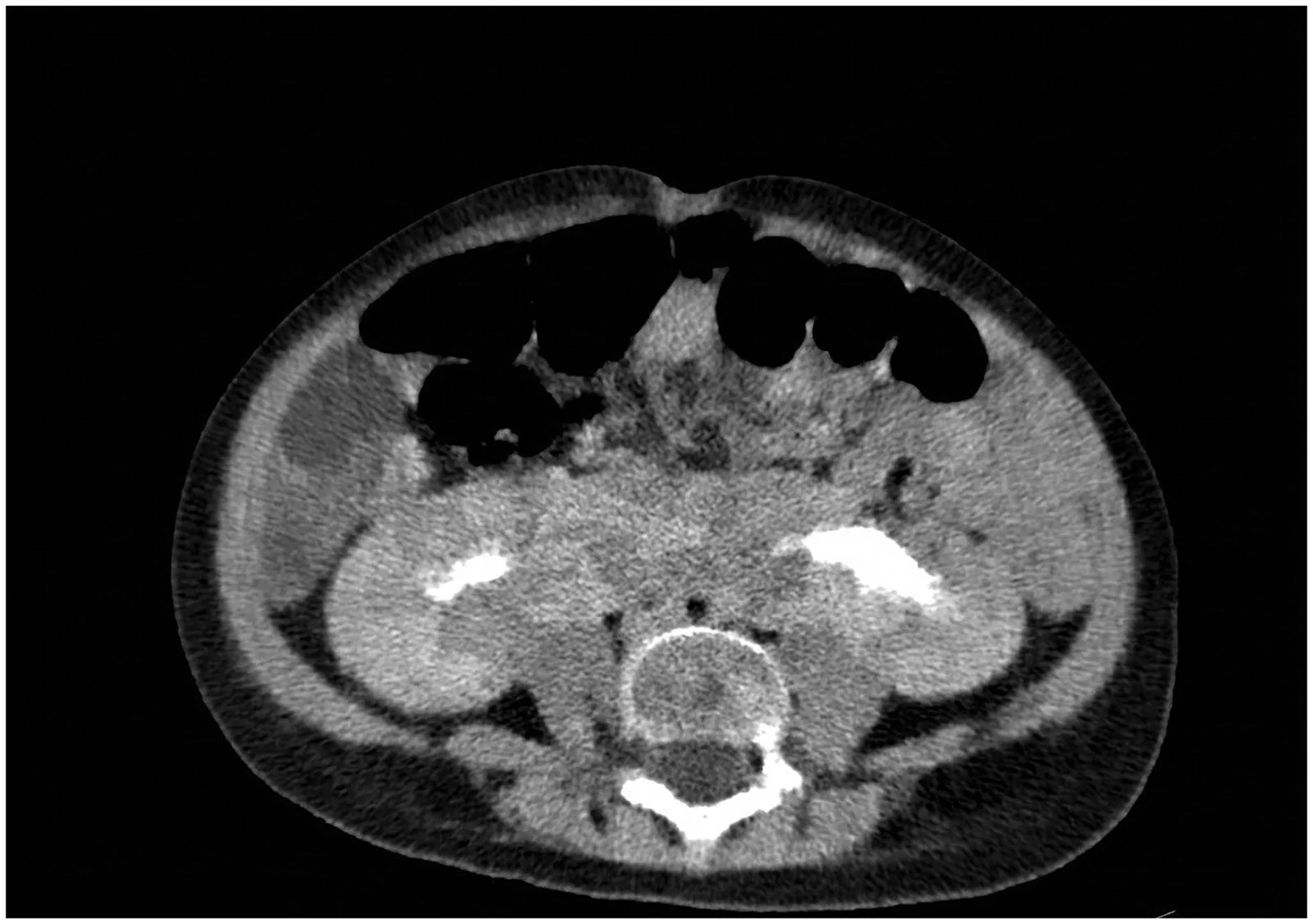
Axial CT scan at the renal level. The scan demonstrates the fusion of the lower poles of both kidneys across the midline anterior to the spine (isthmus), confirming the diagnosis of a horseshoe kidney.

Due to the critical coagulopathy, a liver biopsy was deemed too high-risk. The patient initially received vitamin K and multiple platelet and fresh frozen plasma transfusions, which yielded minimal increment, suggesting peripheral consumption. A bone marrow aspiration at 10 months revealed a normocellular marrow with adequate megakaryocytes, excluding leukemia. A presumptive diagnosis of immune thrombocytopenia (ITP) was made. The patient sequentially received a standard trial of intravenous immunoglobulin (IVIG, 1 g/kg/dose for two days) and a short course of high-dose corticosteroids (prednisolone 2 mg/kg/day for 14 days), with no sustained platelet response. Due to the lack of response and critical bleeding risk, rituximab (375 mg/m^2^ weekly for four doses) was administered as a second-line agent, again without success. A conservative ‘wait and see’ strategy was temporarily adopted.

At 18 months, the patient developed an intracranial hemorrhage. This crisis prompted a metabolic workup, which confirmed Hereditary Tyrosinemia Type 1 (HT-1) via elevated succinylacetone and a homozygous pathogenic variant in the *FAH* gene. Nitisinone (NTBC) was initiated (1 mg/kg/day) alongside a protein-restricted diet.

Over the subsequent 20 months of follow-up, nitisinone therapy resulted in the normalization of liver enzymes and coagulation profile (INR 0.92) and a dramatic decline in AFP to 69 ng/mL. Despite this metabolic control, severe thrombocytopenia (<10 × 10^9^/L) persisted. Secondary autoimmune and infectious etiologies were comprehensively excluded; antinuclear antibodies (ANA), anti-double-stranded DNA (anti-dsDNA), and antiphospholipid antibodies were all negative. Extended viral panels, including human immunodeficiency virus (HIV), Epstein–Barr virus (EBV), and hepatitis B and C, were non-reactive. Furthermore, repeat extensive workup ruled out hypersplenism (spleen 6.3 cm) and primary antibody-mediated deficiency. Whole-exome sequencing identified a heterozygous variant of uncertain significance in ANKRD26; however, repeat bone marrow examination showed normal trilineage hematopoiesis without the dysmegakaryopoiesis typical of ANKRD26-related thrombocytopenia. Subsequently, a trial of the thrombopoietin receptor agonist (TPO-RA) romiplostim was initiated, but the patient proved completely refractory. Future management considerations are severely constrained by the underlying hepatic vulnerability, rendering further experimental immunosuppression highly challenging. She is currently maintained on nitisinone and a restricted diet, clinically stable with only occasional minor gum bleeding.

## Discussion

The coexistence of Hereditary Tyrosinemia Type 1 (HT-1) and refractory Immune Thrombocytopenia (ITP) presents a complex diagnostic and therapeutic dilemma. In HT-1, thrombocytopenia is typically secondary to portal hypertension, hypersplenism, or impaired hepatic synthesis of thrombopoietin. However, our patient demonstrated a distinct dissociation between metabolic control and hematologic status. While nitisinone therapy successfully normalized liver enzymes and coagulation parameters, severe thrombocytopenia persisted. Furthermore, the absence of splenomegaly and the presence of abundant megakaryocytes on bone marrow aspiration strongly excluded liver-related sequestration or production failure, confirming an immune-mediated destruction consistent with ITP [3–5].

The pathophysiology linking these two rare conditions remains speculative but implies a disruption in immune tolerance. In HT-1, the accumulation of toxic metabolites, specifically fumarylacetoacetate and succinylacetone, induces cellular damage and oxidative stress. It is plausible that this chronic metabolic stress promotes the formation of neoantigens or alters immune regulation, triggering an autoimmune response against platelets [6]. While direct autoimmune phenomena are rarely documented specifically in HT-1, chronic liver diseases and profound metabolic derangements of various etiologies are known modulators of immune dysfunction, providing a strong pathophysiological basis for disrupted immune tolerance. Although a variant in *ANKRD26* was identified, the patient’s normocellular marrow lacked the dysmegakaryopoiesis typical of inherited thrombocytopenias [7], reinforcing the diagnosis of acquired autoimmunity. However, while the metabolic dysregulation in HT-1 provides a biologically plausible trigger for autoimmunity, it must be emphasized that this case report demonstrates an association rather than establishing a definitive causal relationship between HT-1 and ITP.

A primary limitation of this report is the lack of histological confirmation via liver biopsy, which was contraindicated due to severe coagulopathy. However, the strength of this case lies in the long-term follow-up (3.5 years) and the robust genetic and metabolic confirmation that clearly delineated the two concurrent pathologies.

An important differential diagnosis in this patient was CMV-induced thrombocytopenia, given the positive CMV PCR. However, active CMV disease was excluded based on several factors: the chronic persistence of severe thrombocytopenia over 3.5 years, the absence of CMV IgM antibodies indicating acute infection, and the total lack of systemic inflammatory signs or end-organ involvement (e.g. retinitis, hepatitis, pneumonitis). Consequently, the positive PCR was attributed to incidental latent viral shedding, a common finding in endemic regions, rather than active viral myelosuppression.

Management of refractory ITP in the setting of HT-1 is particularly challenging. Our patient met the definition of refractory ITP by failing to respond to corticosteroids, IVIG, rituximab, and thrombopoietin receptor agonists (TPO-RAs) [8, 9]. The choice of second-line agents is constrained by the underlying hepatic vulnerability; for instance, TPO-RAs and potential future immunosuppressants carry risks of hepatotoxicity that require rigorous monitoring in a liver already compromised by metabolic disease [10].

## Conclusion

To our knowledge, this case represents the first reported co-occurrence of HT-1 and refractory ITP in the English literature. It highlights that hematologic abnormalities in metabolic disorders should not always be attributed to the primary disease. Clinicians must maintain a high index of suspicion for concurrent autoimmune processes, especially when thrombocytopenia is disproportionate to the degree of liver dysfunction.

## Data Availability

All data relevant to this case report are included in the published article. Any additional information regarding this case report may be obtained by contacting the corresponding author.
